# A Theoretical Study on the Structural Evolution of Ru–Zn Bimetallic Nanoparticles

**DOI:** 10.3390/nano15080568

**Published:** 2025-04-08

**Authors:** Luxin Mu, Jingli Han, Yongpeng Yang

**Affiliations:** 1 Henan Institute of Advanced Technology, Zhengzhou University, Zhengzhou 450003, China; muluxin@gs.zzu.edu.cn; 2 School of Material and Chemical Engineering, Zhengzhou University of Light Industry, Zhengzhou 450001, China; hanj@zzuli.edu.cn

**Keywords:** Ru–Zn bimetallic nanoparticles, machine learning potential, stability, Ru/Zn ratio effect, size effect

## Abstract

Ru–Zn catalysts exhibit excellent catalytic performance for the selective hydrogenation of benzene to cyclohexene and has been utilized in industrial production. However, the structure–performance relationship between Ru–Zn catalysts and benzene hydrogenation remains lacking. In this work, we focused on the evolution of Ru–Zn nanoparticles with size and Ru/Zn ratio. The structures of Ru nanoparticles and Ru–Zn bimetallic nanoparticles with different sizes were determined by the minima-hopping global optimization method in combination with density functional theory and high-dimensional neural network potential. Furthermore, we propose the growth mechanism for Ru nanoparticles and evolution processes for Ru–Zn bimetallic nanoparticles. Additionally, we analyzed the structural stability, electronic properties, and adsorption properties of Zn atoms. This work provides valuable reference and guidance for future theoretical research and applications.

## 1. Introduction

Cyclohexene can be used to synthesize high-value-added chemicals such as cyclohexanol, adipic acid, and caprolactam [1,2], and it is widely utilized, including in medicine, food, dyes, and polyester materials. The selective hydrogenation of benzene to cyclohexene has garnered significant attention compared to other synthesis routes, owing to its inexpensive raw materials, high atom economy, straightforward operation, and environmental friendliness. However, benzene prefers to further hydrogenate to form cyclohexane rather than cyclohexene [1,3,4,5]. Therefore, it has become the focus of research to design suitable catalysts to improve the selectivity and yield of cyclohexene.

Ru has good catalytic properties and can effectively catalyze a variety of chemical reactions, especially in hydrogenation [6,7,8], reduction [9,10], oxidation [11], and Fischer–Tropsch synthesis reactions [12,13]. The relatively low cost of Ru compared to other precious metals makes it more economical in industrial applications. Pure Ru has high activity in the hydrogenation of benzene, but the selectivity to cyclohexene is not as high as expected. Adding other metals or non-metals as auxiliaries can improve the selectivity of cyclohexene. Struijk et al. [14] reported that pure Ru catalyst has a very low cyclohexene yield of only 2%. After adding n−hexane and methanol as additives, the yield increased to 8%. The unsupported Ru-Zn catalyst first developed by Japan’s Asahi Kasei Company significantly increased the cyclohexene selectivity, and its selectivity was over 80% at a benzene conversion of 40% [15]. Liu and Sun et al. conducted extensive research on Ru–Zn catalysts prepared via the co-precipitation method [16,17,18,19]. The Ru–Zn bimetallic catalyst, synthesized using a 10% sodium hydroxide solution as the precipitant, achieved the cyclohexene selectivity of 85.5% at the benzene conversion of 40%.

To investigate the structure–performance relationship between Ru catalysts and benzene hydrogenation, much research has been conducted. Wang et al. [20,21] observed the presence of the Ru(1011) surface in the Ru-based catalyst, noting that the particle size was concentrated between 2 and 4 nm, with the optimal catalyst size being approximately 3 nm. Similarly, Liu et al. [22] identified the presence of the Ru (1011) surface in the Ru–Zn catalyst, which exhibited a particle size of 4.5 nm. Zong et al. [23] observed the presence of the Ru (1012) surface and ZnO (1011) surface in the Ru–Zn catalyst, which exhibited a particle size of 4.2 nm.

He et al. studied the effect of Zn on the benzene hydrogenation in Ru–Zn catalyst through theoretical and experimental methods, which showed that the presence of Zn weakens the adsorption of benzene and cyclohexene [24]. In the middle and late stages of the reaction, the Zn species can also effectively prevent the re-adsorption of cyclohexene, thereby improving the selectivity of cyclohexene. The Zn content in the Ru–Zn catalyst also affects catalytic activity and selectivity. In their work, the optimal Zn content in the catalyst is 2.72%, and the yield of cyclohexene is up to 44%. The optimal Ru/Zn ratio for high cyclohexene selectivity also strongly depends on the synthesis method of Ru–Zn catalysts. The optimal Zn content is 16.7 wt% by using the co-precipitation method, and the catalysts exhibited 80% cyclohexene selectivity at a benzene conversion of 57% [25]. The Ru–Zn (Zn/Ru molar ratio = 0.71) catalysts synthesized by the reaction–adsorption method exhibit 80.6% cyclohexene selectivity at a benzene conversion of 43.6% [20].

Although a lot of research on Ru–Zn bimetallic catalysts for benzene hydrogenation has been conducted, the structure–performance relationship between Ru–Zn catalysts and benzene hydrogenation still needs to be further studied. In this work, we focused on the evolution of Ru–Zn nanoparticles with size and Ru/Zn ratio. Firstly, the pure Ru and Ru–Zn bimetallic nanoparticles with different sizes were confirmed by the minima-hopping global optimization method combined with density functional theory and high-dimensional neural network potential (HDNNP). The distribution pattern of Zn on Ru nanoparticles was confirmed. Lastly, the adsorption properties of Zn atoms on Ru surfaces were studied.

## 2. Computational Details

### 2.1. Density Functional Calculations

All the DFT calculations were performed using CP2K code [26]. The Perdew–Burke–Ernzerhof (PBE) exchange correlation [27] and DZVP molecularly optimized basis sets [28] combined with Goedecker–Teter–Hutter (GTH) pseudopotentials [29] were used. The plane-wave cutoff energy was set at 500 Ry, and the self-consistent field (SCF) convergence was set to be 1.0 × 10^−6^ Ha. The DFT calculation used periodic boundary conditions in XYZ directions. A vacuum thickness of 10 Å was added in the X, Y, and Z directions for the nanoparticles. The Ru surface model consisted of six 6 × 6 Ru atom layers, with the bottom two atomic layers fixed, and the vacuum thickness for the Z direction was set as 10 Å. The Brillouin zone was sampled using the gamma point. For H, C, Zn, and Ru, 1, 4, 12, and 16 valence electrons were considered, respectively. The D3 dispersion correction with Becke–Johnson damping [30] was applied to improve the van der Waals interaction. Geometry optimizations were performed by the Broyden–Fletcher–Goldfarb–Shanno (BFGS) minimization algorithm.

### 2.2. Training of High-Dimensional Neural Network Potential

The generation of training data was mainly based on the minima-hopping method. The minima-hopping method can efficiently sample different structures [31,32], and the temperature information ranging from zero to above melting point was included. For each minima-hopping simulation, about 1000 structures were generated. A total of 56,860 structures were considered, and the nanoparticle size ranges from 55 to 325, including pure Ru, pure Zn, and Ru–Zn bimetallic nanoparticles with face center cubic (FCC) and hexagonal close-packed (HCP) structures. During the HDNNP training, 90% of the data were selected as training data, and the remaining 10% of the data were testing data.

The training of HDNNP was conducted by using open-source n2p2 code [33,34]. The HDNNP consists of two hidden layers, each containing 20 nodes. A total of 400 symmetric functions were used to describe the atomic environment, including 92 radial symmetry functions ($G_{i}^{2}$) and 308 narrow angle symmetry functions ($G_{i}^{3}$). Meanwhile, 204 and 196 symmetry functions were used for Ru and Zn, respectively. The cutoff radius was 14.0 bohr. The form of specific function for HDNNP is shown in S1.

To improve the accuracy of HDNNP, we trained eight HDNNPs by changing the random number generator seed for the committee–HDNNP, presented by Schran et al. [35].

### 2.3. Molecular Dynamics Simulations

The molecular dynamics (MD) simulations with Born–Oppenheimer approximation was performed using a constant-temperature and -volume (NVT) ensemble with the canonical sampling through velocity rescaling (CSVR) thermostats and a time step of 1 fs. MD simulation used cluster boundary conditions, and the vacuum thickness of 10 Å was added in the X, Y, and Z directions.

## 3. Results and Discussion

### 3.1. HDNNP Performance for Ru–Zn

**Figure 1:**
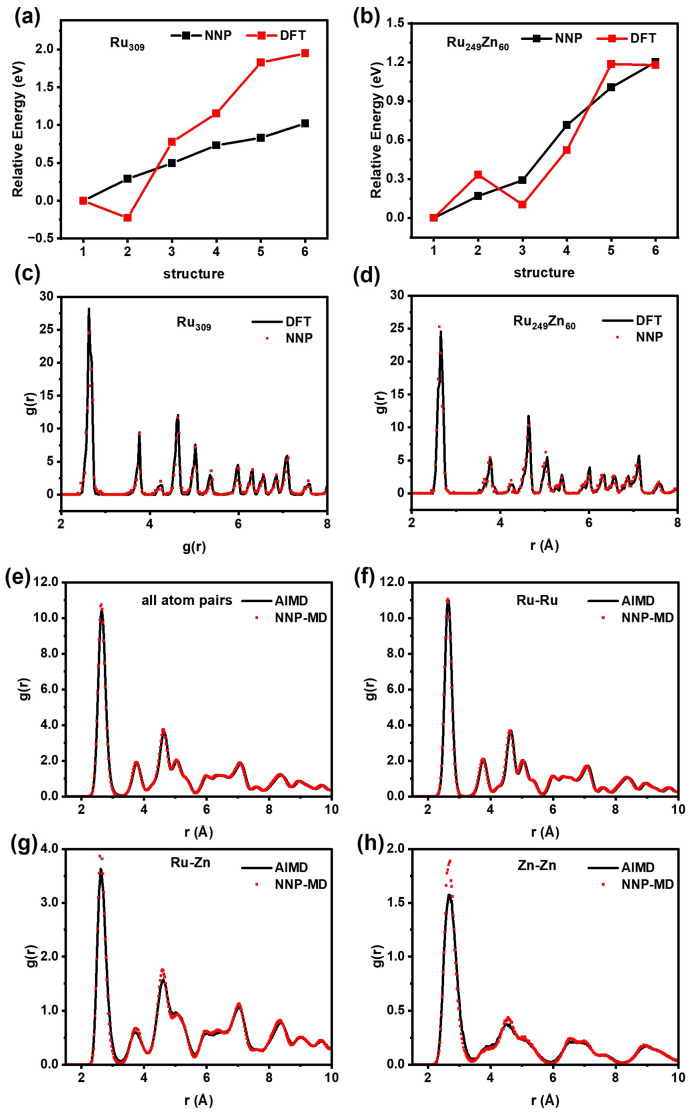
Comparison of HDNNP and DFT. The energy of (**a**) Ru_309_ and (**b**) Ru_249_Zn_60_. The pair radial distribution functions of global minimum structure for (**c**) Ru_309_ and (**d**) Ru_249_Zn_60_. The pair radial distribution functions of MD simulations for Ru_153_Zn_54_ at 700 K for 20 ps, including (**e**) all atom pairs, (**f**) Ru−Ru, (**g**) Ru–Zn, and (**h**) Zn−Zn.

By varying the random seed, eight neural network potentials were obtained for the committee strategy. Table 1 presents the root mean square error (RMSE) for energy and forces. The RMSE for energy ranges from 4.63 to 5.14 meV/atom, while that for forces ranges from 228 to 247 meV/Å. Then, we evaluated the accuracy of HDNNP relative to DFT calculation, as shown in Figure 1. Firstly, the global minimum structures of Ru_309_ and Ru_249_Zn_60_ were searched by using the minima-hopping method combined with committee–HDNNP, and the low-energy structures were further re-optimized by using DFT. It is found that the relative energy calculated using HDNNP is very close to that re-calculated using DFT, and HDNNP can reproduce the geometry structure very well (Figure 1a–d).

Subsequently, MD simulations of Ru_153_Zn_54_ were performed using committee–HDNNP and DFT at 700 K for 20 ps, respectively. The pair radial distribution functions (PRDFs) were analyzed, including all atom pairs, Ru−Ru, Ru–Zn, and Zn−Zn, as shown in Figure 1e–h. All the heights and locations of PRDF peaks obtained by using HDNNP are consistent with those obtained by using DFT very well. The energy trends and atomic distributions predicted by HDNNP align closely with those obtained by using DFT, demonstrating that HDNNP has acceptable accuracy for Ru nanoparticles and Ru–Zn bimetallic nanoparticles from 0 K to high temperature.

### 3.2. Global Minimum Structures of Ru Nanoparticles

The Ru nanoparticles, of which the size is up to 309 atoms, are studied. For smaller Ru particles, 103, 155, 206, and 249 atoms are considered first for the comparison of Ru_n_Zn_309−n_ bimetallic nanoparticles, where the Ru/Zn ratio is 1:2, 1:1, 2:1, and 4:1, respectively. The minima-hopping method was performed for all the nanoparticles by combining with committee–HDNNP, and the low-energy structures were further re-optimized by using DFT to determine the global minimum structure. The various isomers were obtained during global minimum optimization, such as amorphous, FCC, and HCP with different atomic layers. However, all the most stable Ru_n_ (n = 103, 155, 206, 249, and 309) structures have HCP structures.

The calculated results reveal that the most stable structure of Ru_103_ is a five-layer nanoparticle with HCP structure and *C_s_* symmetry. The lowest structure having six-layer structure is 0.63 eV higher in energy, and a seven-layer structure with *D_3h_* symmetry is 2.28 eV higher (Figure S1). For Ru_155_, the most stable configuration is a seven-layer nanoparticle with C*_s_* symmetry. On the other hand, the lowest six-layer structure has adatoms and defects on the (1011) surfaces, and its energy is higher by 0.34 eV. A six-layer structure with only one defect atom is higher by 0.59 eV, and a five-layer structure with *C_s_* symmetry is higher by 2.48 eV (Figure S2), compared with the global minimum of Ru_155_. The most stable structure of Ru_206_ is also a seven-layer nanoparticle with *C_s_* symmetry, and the lowest eight-layer structure with *C_s_* symmetry and the lowest six-layer structure have much higher energies, which are higher by 2.95 eV and 3.05 eV (Figure S3), respectively. Similarly, Ru_249_ still exhibits a seven-layer *C_s_* configuration as the most stable structure, while the lowest eight-layer structure with adatoms and defects on the (1011) surfaces is higher by 1.17 eV. The eight-layer structure with only one adatom is 1.90 eV higher, and the lowest nine-layer *C_s_* structure is 2.09 eV higher (Figure S4). For Ru_309_, the most stable configuration is an eight-layer nanoparticle with *C_1_* symmetry. The lowest seven-layer structure with adatoms and defects on the (1011) surfaces is only higher by 0.29 eV, but the perfect seven-layer structure with *C_2v_* symmetry is higher by 1.07 eV. Meanwhile, the lowest nine-layer structure with *C_2v_* symmetry is higher by only 0.30 eV than the global minimum. As a special crystal configuration, the icosahedral Ru_309_ has much higher energy, which is 7.18 eV higher than the global minimum structure (Figure S5).

**Figure 2:**
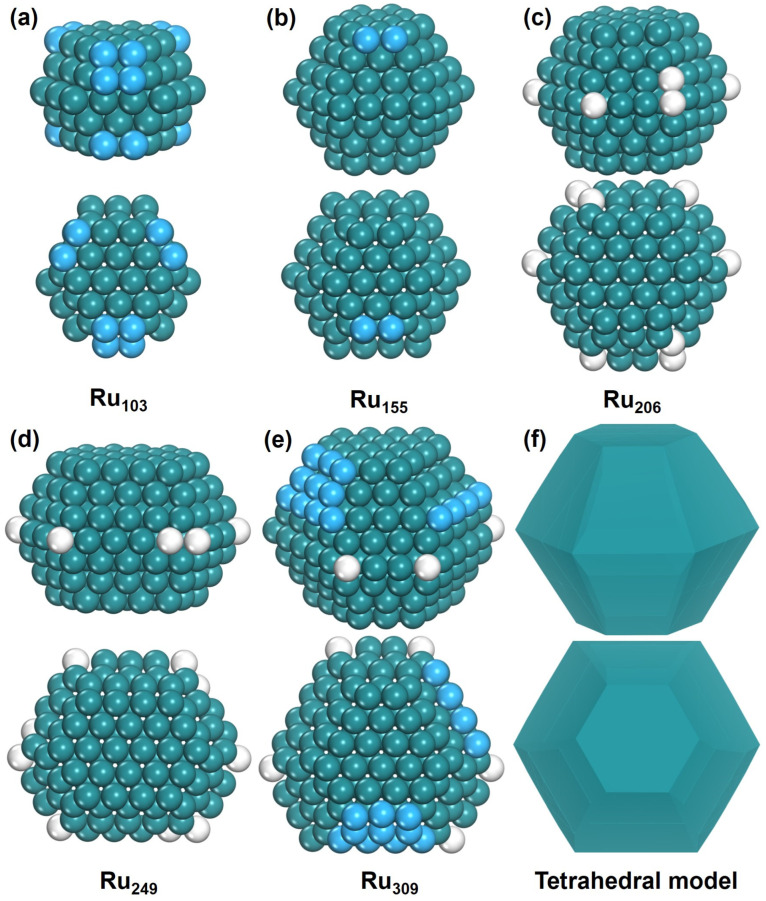
The structure of Ru_n_. Side view and top view of (**a**) Ru_103_, (**b**) Ru_155_, (**c**) Ru_206_, (**d**) Ru_249_, (**e**) Ru_309_, and (**f**) tetrahedral model. The blue ball represents adatom and the white ball represents defect atom.

The most stable structures of Ru_n_ (n = 103, 155, 206, 249, and 309) are shown in Figure 2; they exhibit similar characteristics. They all have approximate tetrahedral HCP-phase configurations, incorporating isolated adatoms and defects. These nanoparticles have hexagonal (0001) surfaces at their top and bottom, while their sides are divided into upper and lower regions, each composed of six (1011) surfaces. Our findings are consistent with relevant experimental and theoretical studies [36,37,38]. Ru_103_ is a five-layer nanoparticle, while Ru_155_, Ru_206_, and Ru_249_ all exhibit seven-layer structures. As the number of atoms increases from 103 to 155, the nanoparticle’s growth occurs on both the (1011) and (0001) surfaces, resulting in an increase in the number of layers. Between Ru_155_ and Ru_249_, its growth primarily occurs on the (1011) surface. From Ru_249_ to Ru_309_, the most stable structures transform into an eight-layer configuration, with atomic growth occurring on both the (1011) and (0001) surfaces.

Many scholars and teams have also studied the nanostructure of Ru. Zhang et al. [39] studied the structure of Ru_n_ (n = 53–58) based on the Wulff construction and proposed that the most stable structure is the FCC-phase structure and compared it with the HCP-phase structure and icosahedral structure. Koyama et al. analyzed the structural stability of the FCC-phase and HCP-phase Ru-NPs with various particle sizes and shapes using DFT. For large sizes, HCP-phase Ru-NPs are the most stable. However, as the number of atoms was less than 103, icosahedral FCC-phase NPs become more stable and dominate.

**Figure 3:**
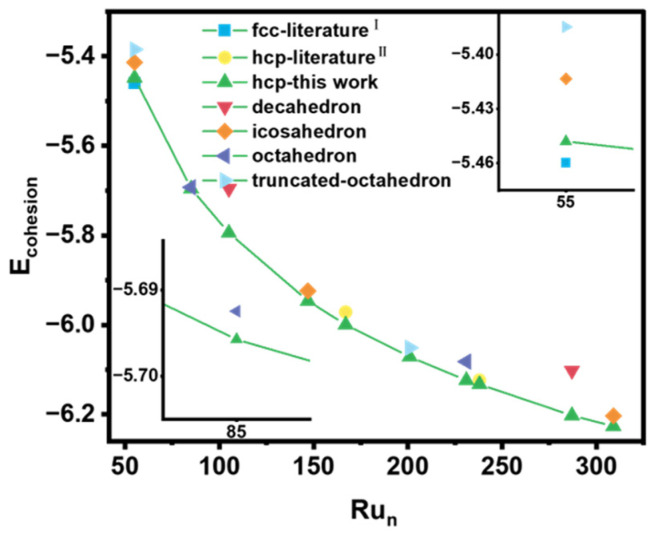
The comparison of the cohesive energy for different Ru cluster configurations.

We compared the structures identified in this work with those reported in the literature and other potential configurations [37,39,40]. The cohesive energies of different Ru cluster configurations are presented in Figure 3. The results show that the HCP-phase structure we proposed is the most stable when the number of atoms exceeds 85. For Ru_n_ (n = 53–58), the FCC-phase structure that Zhang et al. proposed is the most stable. Further, we analyzed the structure of Ru_n_ (n = 59–85). For Ru_84_, the most stable structure is an octahedron with one defect atom, possessing high symmetry with *C_4v_*. The five-layer HCP structure with *C_s_* symmetry for Ru_84_ is 0.26 eV higher in energy, indicating lower stability. For Ru_85_, the most stable configuration is a five-layer HCP structure with *C_s_* symmetry, whereas the octahedral structure with *O_h_* symmetry is 0.28 eV higher in energy (Figure S6). These results indicate a preference for HCP-phase nanoparticles as the dominant structural phase for larger Ru nanoparticles.

When the number of atoms is less than 84, FCC-phase nanoparticles are predominant. However, as the number of atoms increases beyond 84, HCP-phase nanoparticles become more stable and dominate. Based on this analysis, we established the critical value for the stable existence of HCP-phase Ru nanoparticles, which occurs when the number of atoms exceeds 84. This finding provides a clear boundary for the phase preference in Ru nanoparticles and highlights the structural evolution from FCC to HCP phases with increasing particles’ size.

**Figure 4:**
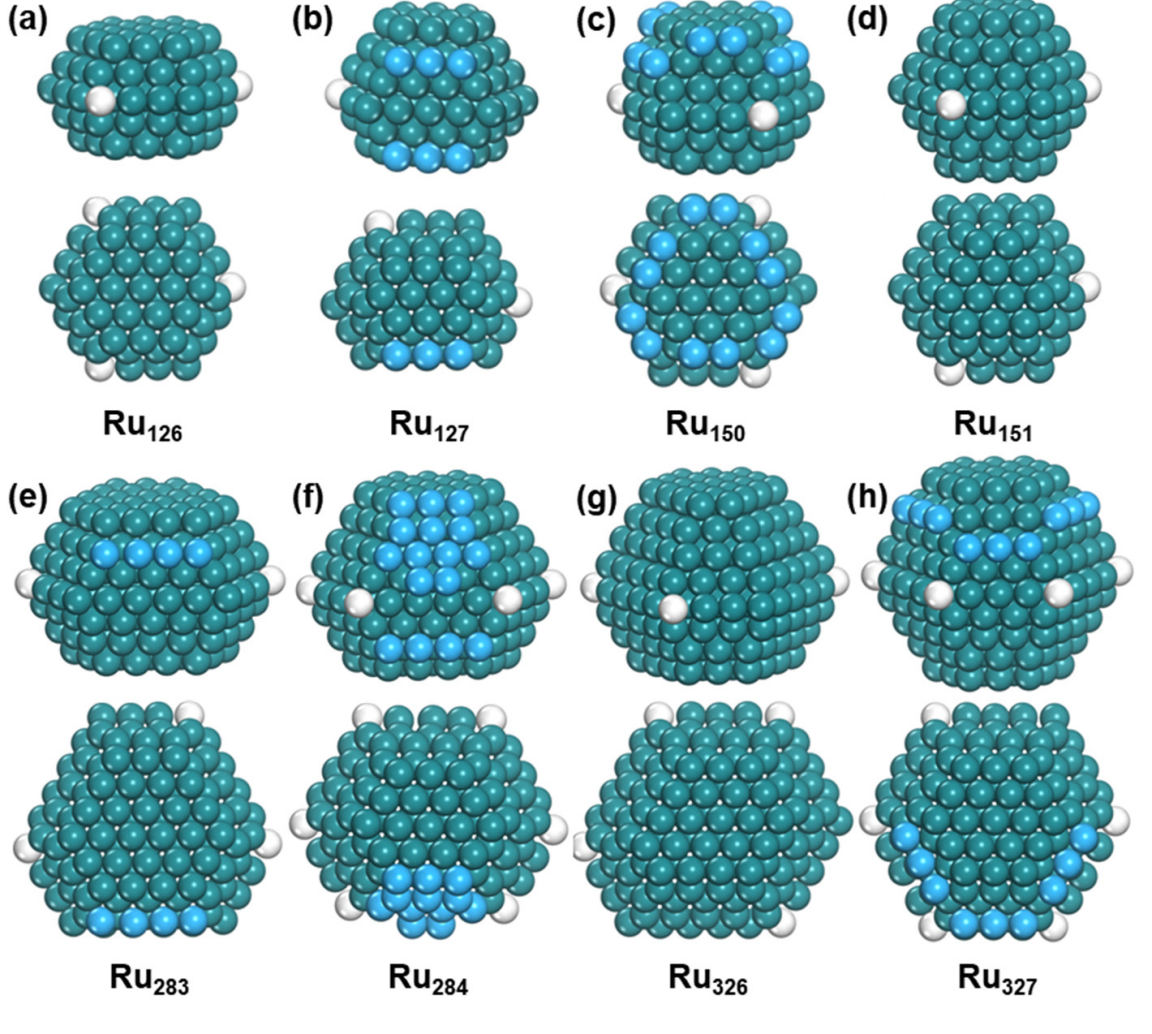
The structure of Ru_n_. Side view and top view of (**a**) Ru_126_, (**b**) Ru_127_, (**c**) Ru_150_, (**d**) Ru_151_, (**e**) Ru_283_, (**f**) Ru_284_, (**g**) Ru_326_, and (**h**) Ru_327_. The blue ball represents adatom and the white ball represents defect atom.

To further investigate the evolution of HCP-phase Ru nanoparticles with size, small nanoparticles (Ru_103_ to Ru_155_) were analyzed. For Ru_126_, the most stable structure is five layers with *C_3h_* symmetry, and the lowest six-layer structure with C_s_ symmetry is higher by 0.22 eV. When the nanoparticle size increases to Ru_127_, its most stable structure changes into a six-layer configuration, and the lowest five-layer structure with *C_s_* symmetry is 0.36 eV higher (Figure S7). For Ru150, the lowest six-layer structure with *C_3_* symmetry and seven-layer structure with *C_3h_* symmetry have very close energy, and the former structure is only 0.07 eV lower than the latter. Finally, Ru_151_ has a more stable seven-layer structure with *C_s_* symmetry, which is 0.15 eV lower in energy than the lowest six-layer configuration (Figure S8). The structural evolution of Ru_n_ is shown in Figure 4.

**Figure 5:**
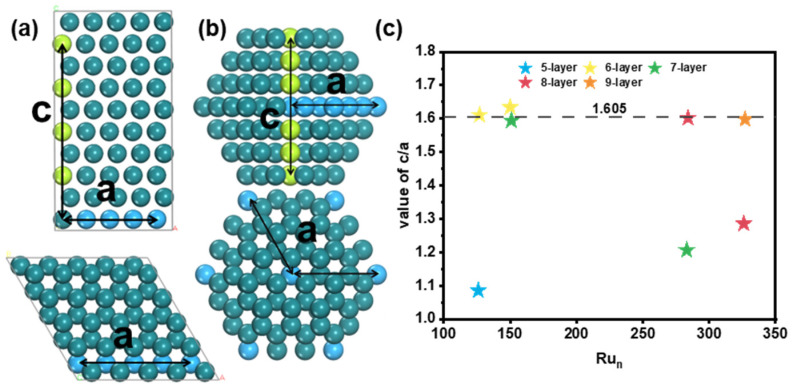
Schematic diagram and comparison for ratio of crystal axes c/a. Schematic diagram of (**a**) Ru bulk, (**b**) Ru cluster, and (**c**) value of c/a for global minimum structures. The blue ball represents atom along the hexagonal base edges, and green ball represents atom along the c-axis.

To study the relationship between the structure and stability of Ru nanoparticles with HCP structures, we introduce a new parameter: the ratio of crystal axes c/a. The parameter a represents the length of the hexagonal base edges, and c represents the height of the unit cell along the c-axis, as shown Figure 5. For bulk Ru, the value of c/a is 1.605. For the most stable Ru_126_ nanoparticle, the c/a value is 1.085, which is significantly smaller than the value of Ru bulk. However, Ru_127_ has a much larger c/a value of 1.611, which is close to the value of Ru bulk, due to the transformation from five layers to six layers. For Ru_150_, which is the largest nanoparticle with a six-layer structure, the c/a value is 1.634, with a lot of adatoms on the six (1011) surfaces. The c/a value is slightly larger than the value of Ru bulk. On the other hand, Ru_151_ is the smallest nanoparticle with a seven-layer structure, and the c/a value is 1.594, slightly smaller than the value of Ru bulk.

From the above results, it is obvious that the c/a value is close to that of Ru bulk for larger nanoparticles. When the c/a value is almost the same as Ru bulk, Ru atoms prefer to grow on the (1011) surfaces. Moreover, the transition of the layer number for larger Ru nanoparticles was further verified. Ru_283_ and Ru_326_ are the biggest nanoparticles with seven-layer and eight-layer structures, respectively, and the c/a value is 1.206 and 1.285, respectively, which are much smaller than the value of Ru bulk. When transforming from seven layers to eight layers for Ru_284_, the c/a value was 1.602, which is close to the value of Ru bulk. Similarly, Ru_327_ is the smallest nanoparticle with a nine-layer structure, of which the c/a value (1.598) is also close to that of Ru bulk (Figures S9 and S10).

### 3.3. The Structural Evolution of Ru–Zn Bimetallic Nanoparticles

**Figure 6:**
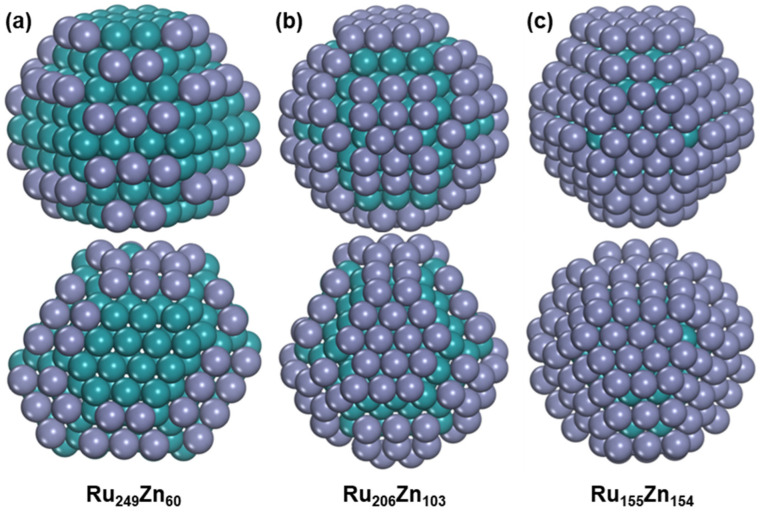
The structure of Ru–Zn bimetallic nanoparticles. (**a**) Ru_249_Zn_60_. (**b**) Ru_206_Zn_103_. (**c**) Ru_155_Zn_154_. The blue-green ball represents Ru atom, and the purple ball represents Zn atom.

Next, the global minimum structures of Ru_n_Zn_309−n_ (n = 249, 206, and 155), corresponding to the Ru/Zn ratios of 4:1, 2:1, and 1:1, were searched by combining the minima-hopping method and HDNNP and confirmed by geometric optimization using DFT. It is found that the Zn atoms prefer to stay at the Ru surface for all the considered Ru_n_Zn_309−n_ bimetallic nanoparticles, which still have HCP structures, as shown in Figure 6. The Ru_249_ nanoparticle changes from seven layers into eight layers after adding sixty Zn atoms, and all the Zn atoms are distributed on the (1011) surfaces. We also found some Ru_249_Zn_60_ nanoparticles where the Ru_249_ structure is the same as the pure Ru_249_, but the lowest structure among them has higher energy by 0.19 eV than the global minimum (Figure S11). With the ratio of Ru/Zn decreasing to 2:1, the Ru_206_ in Ru_206_Zn_103_ still has the original seven-layer structure with some surface Ru atoms being redistributed. Not only the (1011) surfaces but also the two (0001) surfaces are covered by Zn atoms, resulting in the formation of a nine-layer structure. Meanwhile, another isomer of Ru_206_Zn_103_ containing the original structure of pure Ru_206_ is found to be 0.25 eV higher than the global minimum, and the (0001) surfaces are also covered by Zn atoms (Figure S12). For Ru_155_Zn_154_, it still has a nine-layer structure, and almost all the Ru surface is fully covered by Zn. The lowest Ru_155_Zn_154_ structure that contains the original pure Ru_155_ structure is also found to be 0.34 eV higher than the global minimum (Figure S13). Thus, the addition of Zn can slightly change the geometric structures of Ru, but the Ru–Zn bimetallic nanoparticles still have HCP structures under the considered situation. In addition, due to the high cohesive energy between Ru atoms, the Ru atoms prefer to occupy core-like positions, and Zn atoms tend to occupy shell-like positions. On the other hand, the Zn adsorption on the Ru (1011) surface is stronger than on the Ru (0001) surface, so Zn atoms prefer to occupy the Ru (1011) surfaces at first.

The average Ru-Ru bond lengths in Ru_249_Zn_60_, Ru_206_Zn_103_, and Ru_155_Zn_154_ are 2.650, 2.657, and 2.669, respectively, and they are 2.617, 2.643, and 2.660 for Ru–Zn bond lengths, suggesting that high Zn content has a more significant effect on the Ru-Zn bond lengths. The average coordination numbers of Ru−Ru and Ru–Zn in the most stable Ru_249_Zn_60_ nanoparticle are 9.494 and 0.964, respectively, and they are 9.502 and 0.944, respectively in the lowest Ru_249_Zn_60_ that has the original Ru_249_ structure. Similarly, the average coordination number of Ru−Ru is also slightly decreased from 9.379 to 9.359 for Ru_206_Zn_103_ after Ru-Zn bimetal formation, and the transition slightly increases the Ru-Zn interaction by increasing the average coordination number of Ru–Zn from 1.709 to 1.718.

**Figure 7:**
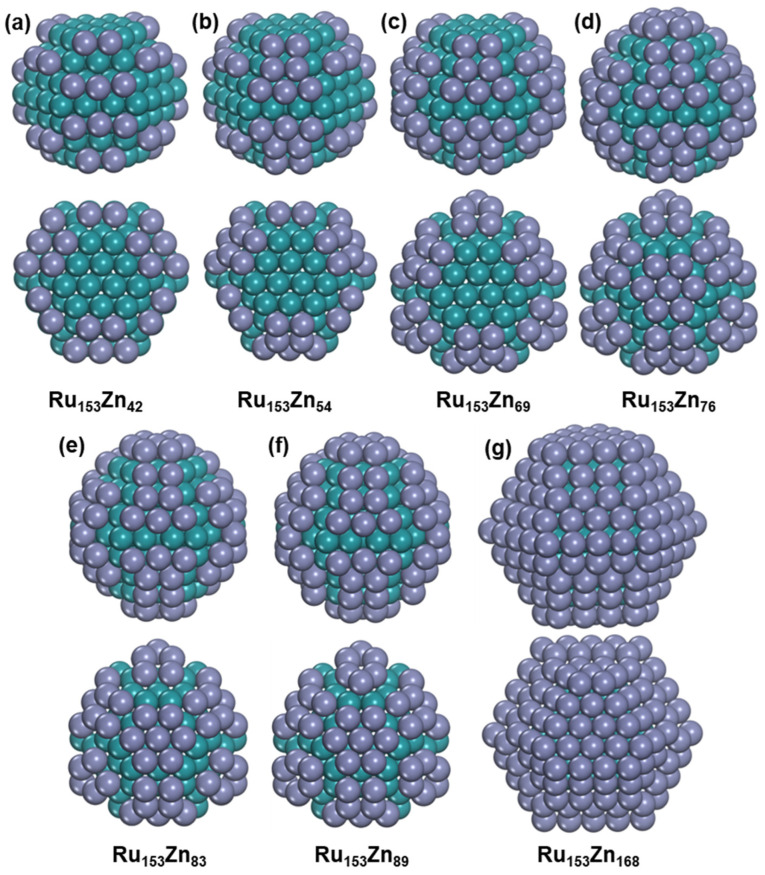
The structure of Ru_153_Zn_x_ bimetallic nanoparticles. (**a**) Ru_153_Zn_42_. (**b**) Ru_153_Zn_54_. (**c**) Ru_153_Zn_69_. (**d**) Ru_153_Zn_76_. (**e**) Ru_153_Zn_83_. (**f**) Ru_153_Zn_89_. (**g**) Ru_153_Zn_168_. The blue-green ball represents Ru atom, and the purple ball represents Zn atom.

In addition, we also studied the growth of Zn on Ru nanoparticles with a fixed Ru atom number, and Ru_153_ is selected. The reason for selecting Ru_153_ is that the Ru_153_ is assumed to have perfect HCP structure without adatoms and defects according to the rule of Ru nanoparticles’ size increasing [37], although the perfect HCP structure of Ru_153_ is found to be only 0.09 eV higher than the global minimum, which contains surface adatoms and defects (Figure S14). Various Zn concentrations were considered for Ru_153_Zn_n_, and all the Zn atoms prefer to be distributed on the Ru surface. We will discuss the most stable structures of Ru_153_Zn_n_ (n = 42, 54, 69, 76, 83, 89, and 168) due to the distinct distribution of Zn, as shown in Figure 7. After adding Zn to Ru_153_, Ru_153_ changes into a perfect HCP structure without any Ru adatoms and defects for all the most stable Ru_153_Zn_n_ (n = 42, 54, 69, 76, 83, 89, and 168) nanoparticles.

Ru_153_Zn_42_ exhibits *D_3__ℎ_* symmetry, with the Zn atoms occupying all fourfold coordination sites on (1011) surfaces of Ru_153_. Similarly, Ru_153_Zn_54_ also displays *D_3__ℎ_* symmetry, and the FCC sites between the fourfold coordination sites; in addition, the fourfold coordination sites on (1011) surfaces of Ru_153_ are occupied by Zn. Ru_153_Zn_69_, which retains *D_3h_* symmetry, has the highest Zn concentration among the considered Ru_153_Zn_n_ nanoparticles, where all the Zn atoms only occupy the (1011) surfaces of Ru_153_. With the content of Zn increasing, the Zn atoms begin to occupy the (0001) surface, although all the (1011) surfaces are not fully covered by Zn. Ru_153_Zn_76_ exhibits *C_3v_* symmetry and has the highest Zn concentration in Ru_153_Zn_n_, where all the Zn atoms occupy one (0001) surface and all (1011) surfaces of Ru_153_. With the content of Zn increasing, the Zn atoms begin to occupy another (0001) surface in Ru_153_Zn_83_ with *D_3__ℎ_* symmetry. All the threefold coordinated sites and the fourfold coordination sites of the Ru surface are occupied by Zn atoms in Ru_153_Zn_89_, and it also exhibits *D_3h_* symmetry. Furthermore, all the surfaces are fully covered by Zn atoms to form a Ru@Zn core–shell structure in Ru_153_Zn_168_ with *D_3h_* symmetry.

The Zn atoms exhibit a consistent distribution in Ru_n_Zn_309−n_ and Ru_153_Zn_x_, with the Zn/Ru ratio increasing, and they initially occupy the fourfold coordination and defect sites on the Ru (1011) surface. Subsequently, they occupy the sites between the fourfold coordination locations and occupy the (0001) surface in sequence.

**Figure 8:**
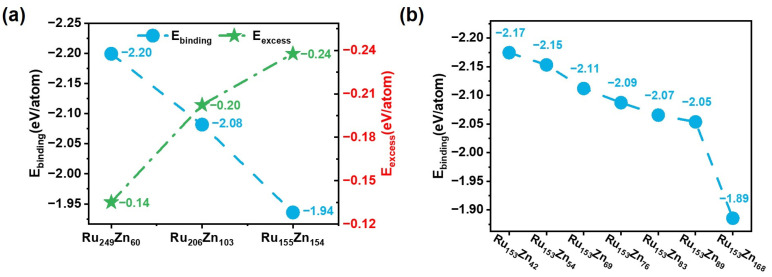
The structural properties of Ru-Zn bimetallic nanoparticles. (**a**) The average binding energy of Zn atoms and the average excess energy for Ru_x_Zn_309−x_. (**b**) The average binding energy of Zn atoms for Ru_153_Zn_n_.

Then, we used average excess energy and the average binding energy of Zn atoms to study the structural stability of Ru-Zn bimetallic nanoparticles. As shown in Figure 8a, the average excess energy of Ru_249_Zn_60_, Ru_206_Zn_103_, and Ru_155_Zn_154_ is −0.14 eV/atom, −0.20 eV/atom, and −0.24 eV/atom, respectively. The negative energies indicate that the considered Ru-Zn bimetallic nanoparticles are more stable than the pure Ru and Zn nanoparticles. On the other hand, the average binding energy of Zn atoms is −2.20 eV/atom, −2.08 eV/atom, and −1.94 eV/atom, suggesting the Ru-Zn interaction is weakened as the Zn/Ru ratio increases.

For Ru_153_Zn_x_, the average binding energies of Zn were also calculated to analyze their structural stability (Figure 8b). The Zn atoms fully occupying all the fourfold coordination sites of Ru (1011) in Ru_153_Zn_42_ have the largest average binding energy (−2.17 eV/atom). Then, it decreases monotonously to −2.15 eV/atom, −2.11 eV/atom, −2.09 eV/atom, −2.07 eV/atom, −2.05 eV/atom, and −1.89 eV/atom for Ru_153_Zn_x_ (x = 54, 69, 76, 83, 89, and 168). Furthermore, MD combined with HDNNP simulations for 1 ns at various temperatures was carried out to study the kinetic stability of Ru-Zn bimetal [41]. As the temperature increases, the diffusion of Zn atoms occurs, and the higher Zn content, the lower the temperature at which Zn diffusion is observed. For example, the Zn diffusion from the Ru (1011) surface to the Ru (0001) surface is observed at 650 K for Ru_153_Zn_42_. However, for Ru_153_Zn_x_ (x = 54, 69, and 76), the Zn diffusion is observed at a lower temperature of 600 K. This fact can be ascribed to the weakened Ru-Zn interaction at higher Zn content, coinciding with the smaller average binding energy of Zn.

### 3.4. The Electronic Properties of Ru Nanoparticles and Ru–Zn Bimetallic Nanoparticles

**Figure 9:**
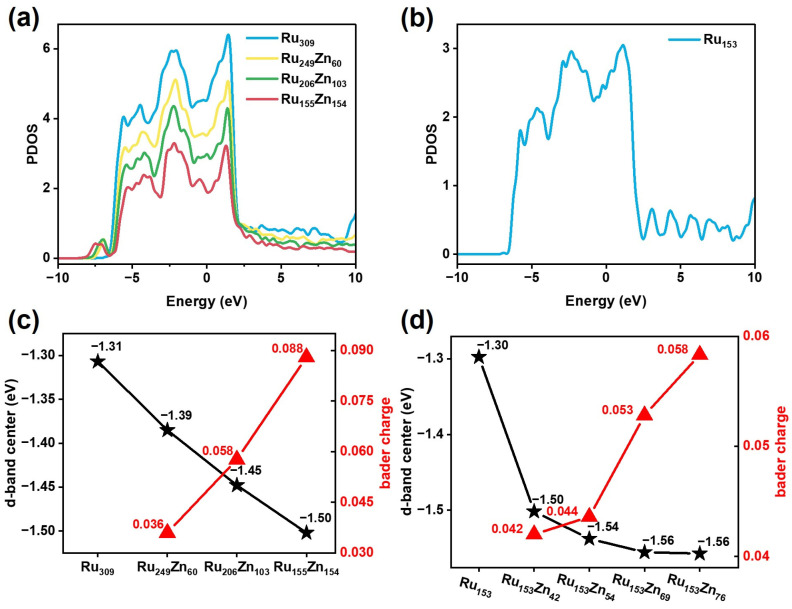
The electronic properties of Ru and Ru–Zn bimetallic nanoparticles. (**a**) The PDOS of d orbitals for (**a**) Ru_n_Zn_309−n_ and (**b**) Ru_153_. The center of the d-band and the transform number of Bader charge for (**c**) Ru_n_Zn_309−n_ and (**d**) Ru_153_Zn_x_.

The evolution of Ru–Zn bimetallic nanoparticles also leads to changes in electronic properties. The electronic properties of Ru_n_Zn_309−n_ and Ru_153_Zn_x_ are shown in Figure 9.

The partial density of states (PDOS) of d orbitals [42] for Ru_n_Zn_309−n_ is shown in Figure 9a. The PDOS shows a similar distribution of electrons, and it is continuous from −10 eV to 10 eV. The center of the d-band for Ru_309_, Ru_249_Zn_60_, Ru_206_Zn_103_, and Ru_155_Zn_154_ is −1.31 eV, −1.39 eV, −1.45 eV, and −1.50 eV (Figure 9c). The center of the d-band is far away from the Fermi level with the proportion of Zn increasing. The electronegativity of the Ru element is higher than that of the Zn element. In the Ru–Zn bimetallic structure, electrons are transferred from Zn to Ru, and the electrons gather on Ru to become negatively charged. The average number of Bader charge transfers per Ru atom for Ru_249_Zn_60_, Ru_206_Zn_103_, and Ru_155_Zn_154_ are 0.036, 0.058, and 0.088. As the proportion of Zn increases, the transfer number of Bader charge increases from Zn to Ru.

For Ru_153_Zn_x_, the PDOS of d orbitals is shown in Figure 9b and Figure S15; it is continuous from −10 eV to 10 eV. The PDOS shifts to the low-energy direction as the content of Zn increases. The center of the d-band for Ru_153_, Ru_153_Zn_42_, Ru_153_Zn_54_, Ru_153_Zn_69_, and Ru_153_Zn_76_ is −1.30 eV, −1.50 eV, −1.54 eV, −1.56 eV, and −1.56 eV (Figure 9d). The average transform number of Bader charge per Ru atom for Ru_153_Zn_42_, Ru_153_Zn_54_, Ru_153_Zn_69_, and Ru_153_Zn_76_ is 0.042, 0.044, 0.053, and 0.058. As the proportion of Zn increases, the transfer number of Bader charge also increases from Zn to Ru. The charge transfer from Zn to Ru leads to the shift of the d-band center of Ru to lower energy, which weakens the interaction between adsorbates and Ru surface. This fact means that the intermediates, such as C_6_H_10_, can more easily be desorbed before further hydrogenated during benzene hydrogenation and increases the selectivity of C_6_H_10_.

### 3.5. The Adsorption Properties of Zn Atoms on Ru Surfaces

In theoretical calculations, specific crystal planes are usually selected to study the reaction performance. We selected the three crystal planes of Ru to study their structure and properties, including the (0001), (1011), and (1019) surfaces (Figure S16). The HCP phase is the most stable crystal form of Ru, and the Ru cluster consists of the (0001) and (1011) surfaces. The (0001) surface is the close-packed crystal plane of the HCP phase, which is often selected for theoretical calculation. The (1011) surface was characterized in the Ru and Ru–Zn bimetallic catalyst [20,22,36]. The stepped structure is a planar dislocation that forms on the surface of a grain during crystal growth, which is common in some metals and their oxides. Experiments have proved the existence of a step structure and can be used to calculate the step height and density [43,44]. The (1019) surface is the stepped plane of Ru, which belongs to the high Miller index crystal plane; John T. Yates and H.J Jänsch studied the adsorption of N_2_ and CO on the (1019) surface, which exhibits a unique structure and chemical properties [45,46].

**Figure 10:**
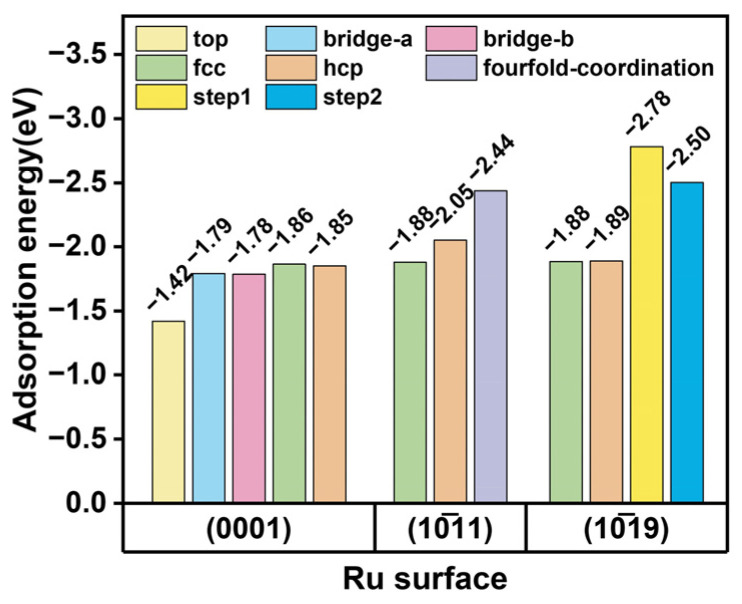
The adsorption of Zn atoms on Ru surfaces.

The adsorption energy of the Zn atom on the Ru surface model has been calculated, with the results presented in Figure 10. Zn atoms are adsorbed at various sites on the (0001) surface; the adsorption energies at the top, bridge a, and bridge b sites are −1.42 eV, −1.79 eV, and −1.78 eV, respectively. The highest adsorption energies are demonstrated at the FCC and HCP sites, which are 1.85 eV and −1.86 eV, respectively. On the (1011) surface, the adsorption energies for the FCC and HCP sites are −1.88 eV and −2.06 eV, respectively, and the strongest adsorption is observed at the fourfold coordination sites, with an adsorption energy of −2.44 eV. In addition, the adsorption energy at various sites on this surface is higher compared to that on the (0001) surface; this observation also elucidates the preferential adsorption of Zn on the (1011) surface within the Ru–Zn bimetal. The (1019) step surface is composed of a terrace and step position; the adsorption energies for the terrace FCC and HCP sites are consistent with those observed on the (0001) surface, measuring −1.88 eV and −1.89 eV, respectively. Furthermore, the step sites exhibit higher adsorption energies, reaching −2.78 eV and −2.50 eV for step1 and step2, respectively. Zn atoms are preferentially adsorbed at the step position. When Zn is introduced, Zn preferentially protects the step position, exposing the (0001) surface; it exhibits similar structural properties to Ru nanoparticles.

## 4. Conclusions

In this study, we determined the structures of Ru nanoparticles and Ru–Zn bimetallic nanoparticles by the minima-hopping global optimization method in conjunction DFT and HDNNP. For Ru_n_ (n = 53–84), it exhibits an FCC-phase structure. As the number of atoms increases beyond 84, Ru nanoparticles exhibit an approximate tetrahedral HCP-phase structure composed of the (0001) and (1011) surfaces. While bimetallic nanoparticles display an HCP-phase Ru@Zn core–shell structure. We propose a growth mechanism for Ru nanoparticles and evolution processes for Ru–Zn bimetallic nanoparticles. Specifically, Ru nanoparticles grow along the (1011) surface; when the c/a value almost the same as Ru bulk, Ru atoms prefer to grow on the (0001) surfaces. In Ru–Zn bimetallic nanoparticles, Zn atoms initially occupy the fourfold coordination and defect sites on the Ru (1011) surface. Subsequently, they occupy the sites between the fourfold coordination locations and occupy the (0001) surface in sequence. Additionally, we analyzed the structural stability and electronic properties; they are closely related to structural evolution. Eventually, we calculated adsorption properties of Zn atoms on Ru surfaces. The Ru (1011) surface shows the preferential adsorption of Zn atoms compared to the (0001) surface. It can be seen from the adsorption properties that the Ru (1019) surface exhibits similar structural properties for Zn to Ru nanoparticles.

This work also serves as a reference and provides guidance for future research and applications:

(1) This work serves as a reference for resolving other metallic nanostructures and studying the evolutionary processes.
(2) It offers insights into directional growth and precise control of Ru nanoparticles and Ru–Zn bimetallic nanoparticles.
(3) It also provides the possibility to study the reactivity of Ru nanoparticles and Ru–Zn bimetallic nanoparticles and reveal the relationship between structure and catalytic performance.

## Figures and Tables

**Table 1:** The RMSE of energy and force.

	Energy RMSE (meV/atom)		Force RMSE (meV/Å)	
	Training	Test	Training	Test
1	4.63	4.77	228	228
2	4.89	5.06	247	247
3	5.01	5.14	241	241
4	4.67	5.06	237	242
5	4.98	5.08	242	243
6	4.80	4.81	247	246
7	4.81	4.92	232	233
8	4.63	4.77	231	232

## Data Availability

The original contributions presented in this study are included in the article/Supplementary Materials. Further inquiries can be directed to the corresponding author.

## Supplementary Materials

The following supporting information can be downloaded at: https://www.mdpi.com/article/10.3390/nano15080568/s1. S1: The form of specific function for HDNNP. S2: The calculation formula. Figure S1: The structure of Ru_103_. Figure S2: The structure of Ru_155_. Figure S3: The structure of Ru_206_. Figure S4: The structure of Ru_249_. Figure S5: The structure of Ru_309_. Figure S6: The structure of Ru_84_ and Ru_85_. Figure S7: The structure of Ru_126_ and Ru_127_. Figure S8: The structure of Ru_150_ and Ru_151_. Figure S9: The structure of Ru_283_ and Ru_284_. Figure S10: The structure of Ru_326_ and Ru_327_. Figure S11: The structure and structural properties of Ru_249_Zn_60_. Figure S12: The structure and structural properties of Ru_206_Zn_103_. Figure S13: The structure and structural properties of Ru_155_Zn_154_. Figure S14: The structure of Ru_153_. Figure S15: The PDOS of d orbitals for (a) Ru_153_Zn_42_, (b) Ru_153_Zn_54_, (c) Ru_153_Zn_69_, and (d) Ru_153_Zn_76_. Figure S16: The Ru surface model. The green and blue balls both represent Ru atoms. Side view and top view (a) Ru (0001), (b) Ru (1011), and (c) Ru (1019) surface model. Figure S17: The change in energy and temperature over time in the AIMD simulation.
