# Supplementary material for: A Theoretical Study on the Structural Evolution of Ru–Zn Bimetallic Nanoparticles
Source: Nanomaterials (Basel). 2025 Apr 8;15(8):568. doi: 10.3390/nano15080568 (PMC12029503; doi:10.3390/nano15080568)
Supplement: Supplementary file 1 [file nanomaterials-15-00568-s001.zip › nanomaterials-3549970-supplementary.pdf]

A Theoretical Study on the Structural Evolution of Ru–Zn Bimetallic Nanoparticles

Luxin Mu [1], Jingli Han [2] and Yongpeng Yang [1,*]

[1] Henan Institute of Advanced Technology, Zhengzhou University, Zhengzhou 450003, China; muluxin@gs.zzu.edu.cn
[2] School of Material and Chemical Engineering, Zhengzhou University of Light Industry, Zhengzhou 450001, China; hanj@zzuli.edu.cn
* Correspondence: ypyang2017@zzu.edu.cn

S1. The form of specific function for HDNNP

The radial symmetry function (G_i^2) and angle symmetry functions (G_i^3) are as follows:

$$G_i^2 = \sum_{j \neq i} \mathrm{e}^{-\eta (r_{ij}-r_s)^2} f_c(r_{ij})$$

$$G_i^3 = 2^{1-\zeta} \sum_{\substack{j,k \neq i \\ j<k}} (1 + \lambda \cos \theta_{ijk})^{\zeta} \mathrm{e}^{-\eta ((r_{ij}-r_s)^2 + (r_{ik}-r_s)^2 + (r_{jk}-r_s)^2)} f_c(r_{ij}) f_c(r_{ik}) f_c(r_{jk})$$

r_{ij}, r_{ik}, r_{jk}represents the distance between atoms i and j, i and k, j and k. θ_{ijk} is the angle composed by the atoms i, j and k, η is proportional constant. For the angular symmetry function, the r_s value is zero in this work, and λ equals to 1 or -1.

where f_c is cutoff function, and

$$f_c(\mathrm{r}) = \tanh^3 \left(1 - \frac{r}{r_c}\right)$$

$$\tanh(x) = \frac{e^x - e^{-x}}{e^x + e^{-x}}$$

r is the distance between atoms, r_c is the truncation radius.

The symmetry function scaling is as follows:

$$G_{\text{scaled}} = S_{\text{min}} + (S_{\text{max}} - S_{\text{min}}) \cdot \frac{G - \langle G \rangle}{G_{\text{max}} - G_{\text{min}}}$$

G is the value of the primary data, $\langle G \rangle$ is the average value, G_{max} is the maximum value, G_{min} is the minimum value, S_{max} is the maximum value of the scaled target range, S_{min} is the minimum value of the scaled target range, G_{scaled} is the result after scaling.

The activation functions of the hidden layer and the output layer are set to TANH function and the linear function, respectively, as follows:

$$f_a(x) = \tanh(\mathrm{x})$$

$$f_a(x) = x$$

S2. The calculation formula

The average excess energy of Ru_xZn_{309-x} is defined as follows:

$$E_{\text{excess}}(\mathrm{Ru}_x\mathrm{Zn}_{309-x}) = \frac{E(\mathrm{Ru_x Zn_{309-x}}) - \frac{x}{309} E(\mathrm{Ru_{309}}) - \frac{309-x}{309} E(\mathrm{Zn_{309}})}{309}$$

where x is the number of Ru atoms in Ru_xZn_{309-x}, $E(\mathrm{Ru_x Zn_{309-x}})$, $E(\mathrm{Ru_{309}})$ and $E(\mathrm{Zn_{309}})$ are the energy of Ru_xZn_{309-x}, Ru_{309} and Zn_{309}, respectively.

The average binding energy of Zn atoms in Ru_mZn_n is defined as follows:

$$E_{\text{bingding}}(\mathrm{Ru}_m\mathrm{Zn}_n) = \frac{E(\mathrm{Ru}_m\mathrm{Zn_n}) - E(\mathrm{Ru}_m) - nE_{\text{atom}}\mathrm{Zn}}{n}$$

where m and n are the number of Ru and Zn atoms in Ru_mZn_n, $E(Ru_mZn_n)$, $E(Ru_m)$ and $E_{atom}Zn$ are the energy of Ru_mZn_n, Ru_m and the single–point energy of the Zn atom.

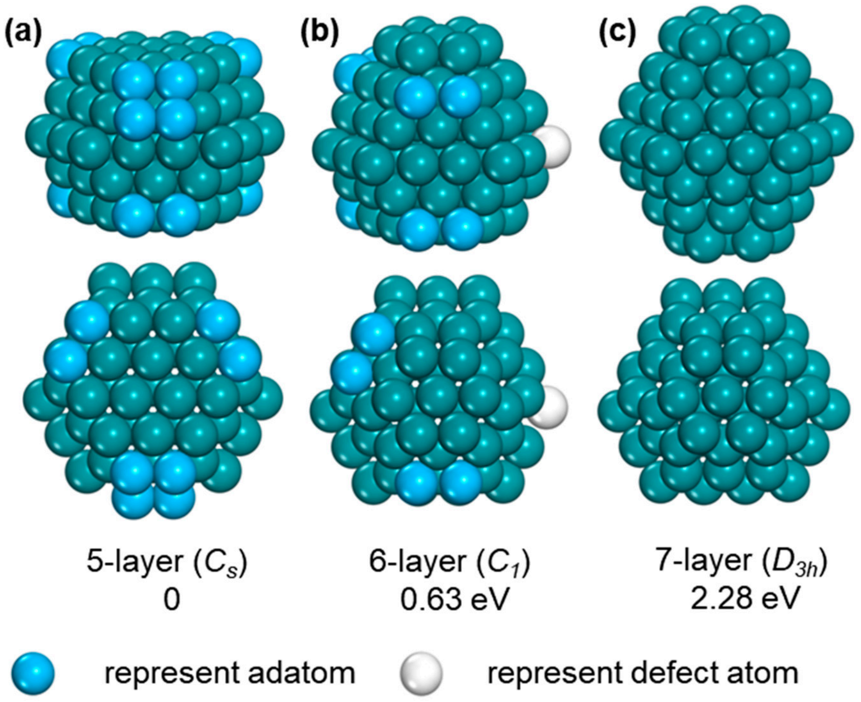

Figure S1. The structure of Ru_{103}.

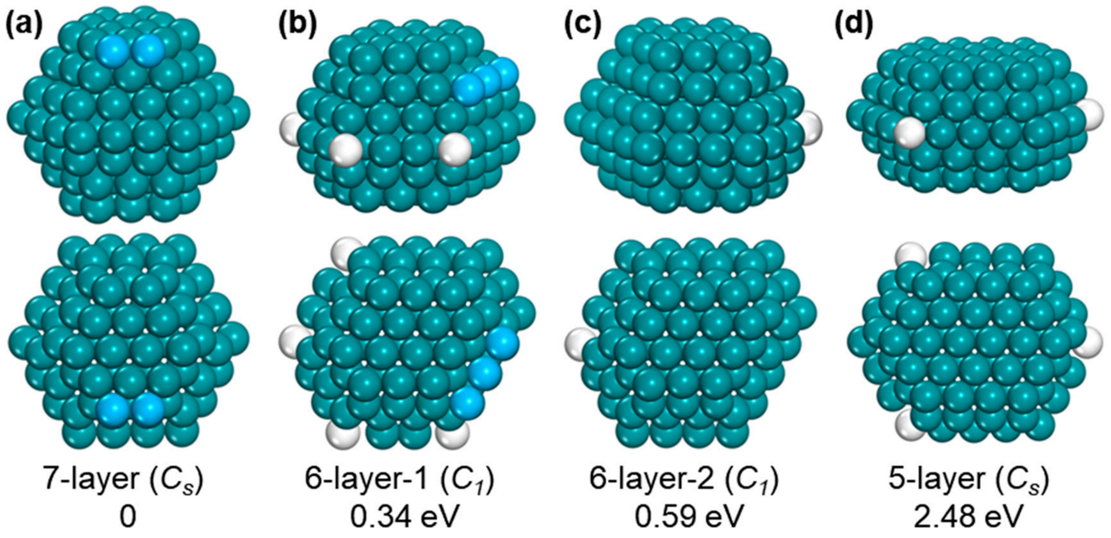

Figure S2. The structure of Ru_{155}.

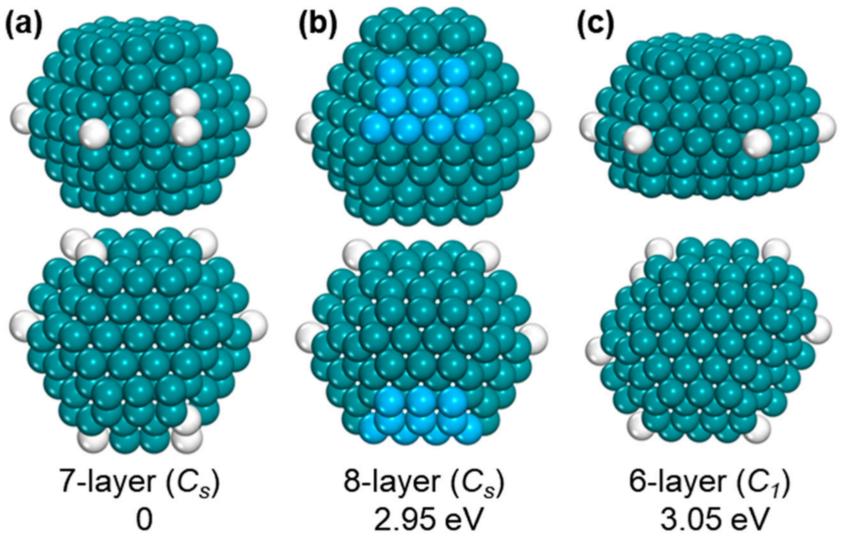

Figure S3. The structure of Ru_{206}.

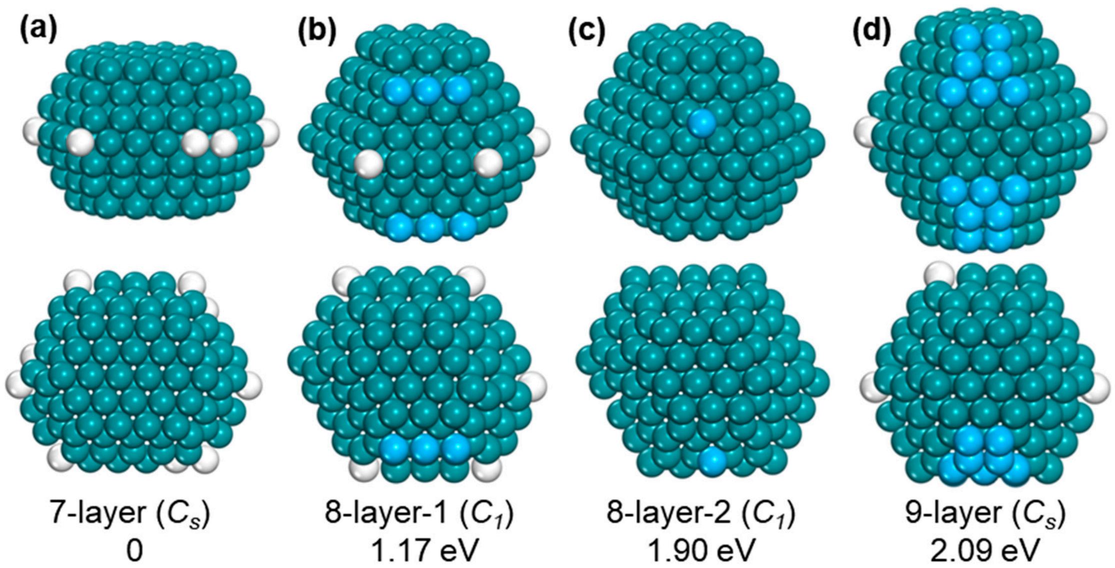

Figure S4. The structure of Ru_{249}.

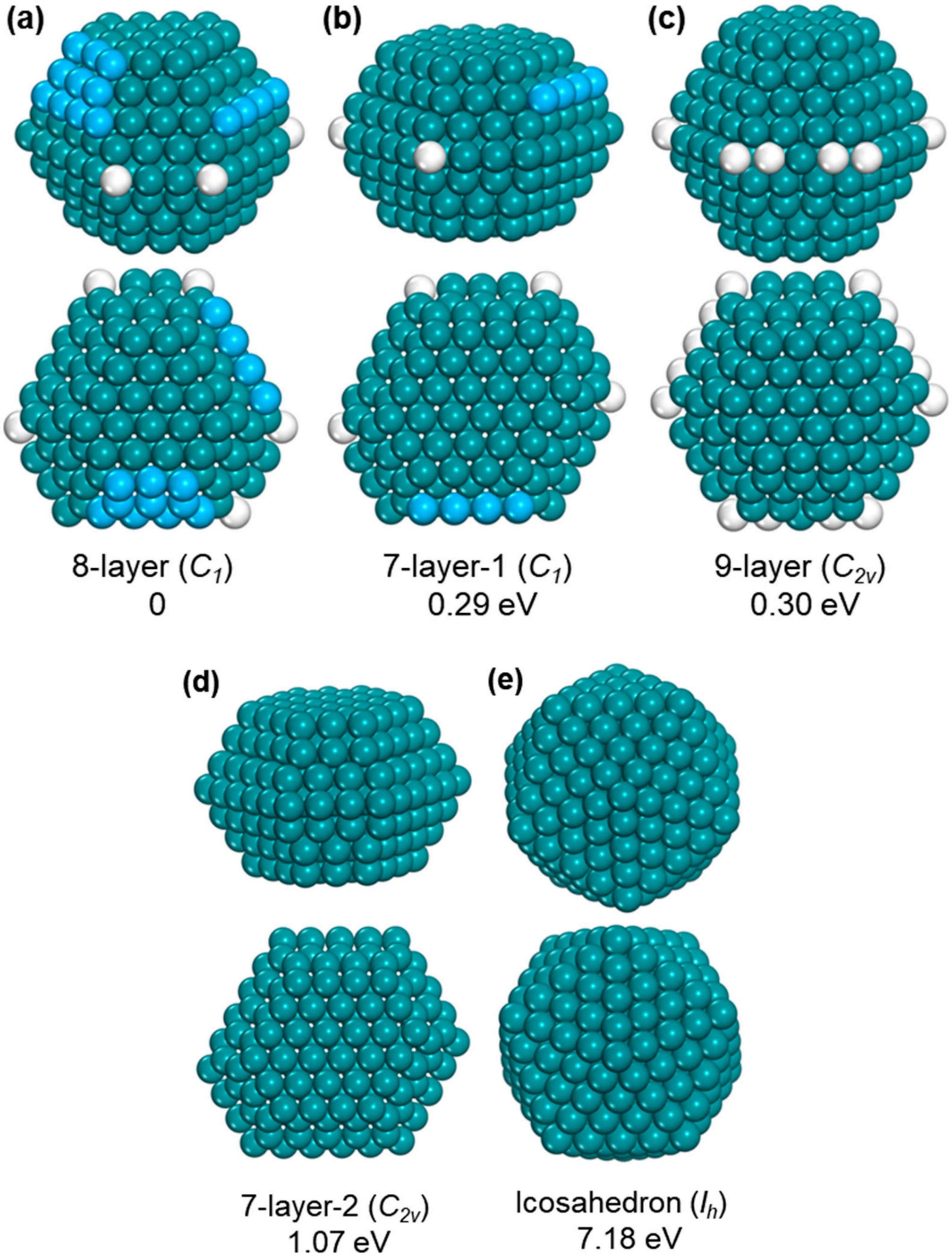

Figure S5. The structure of Ru_{309}.

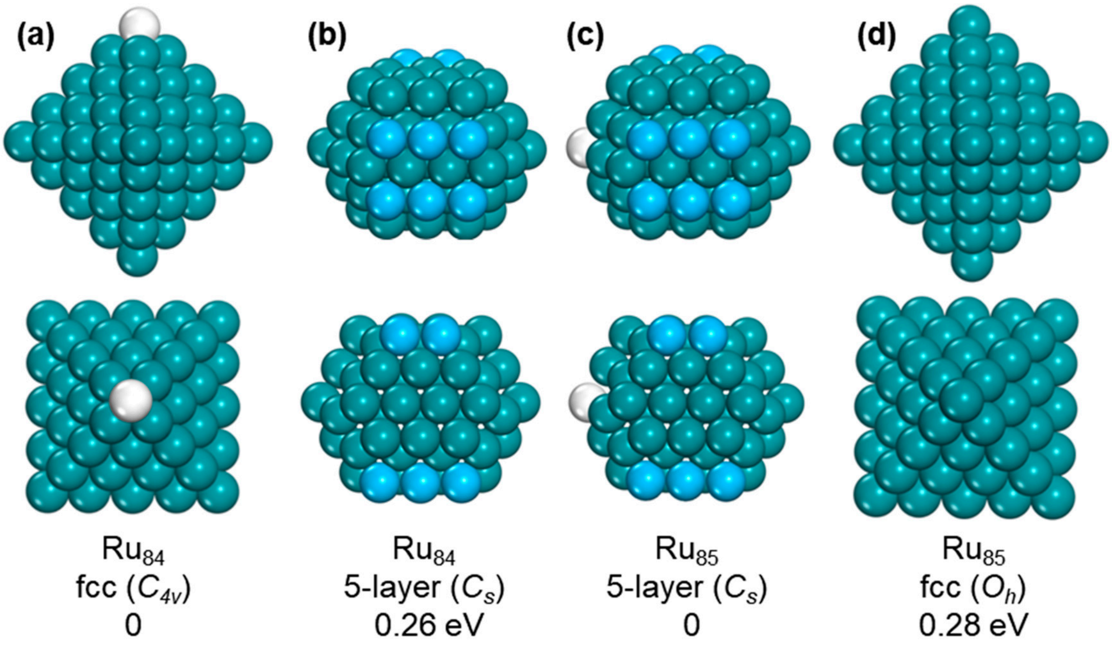

Figure S6. The structure of Ru_{84} and Ru_{85}.

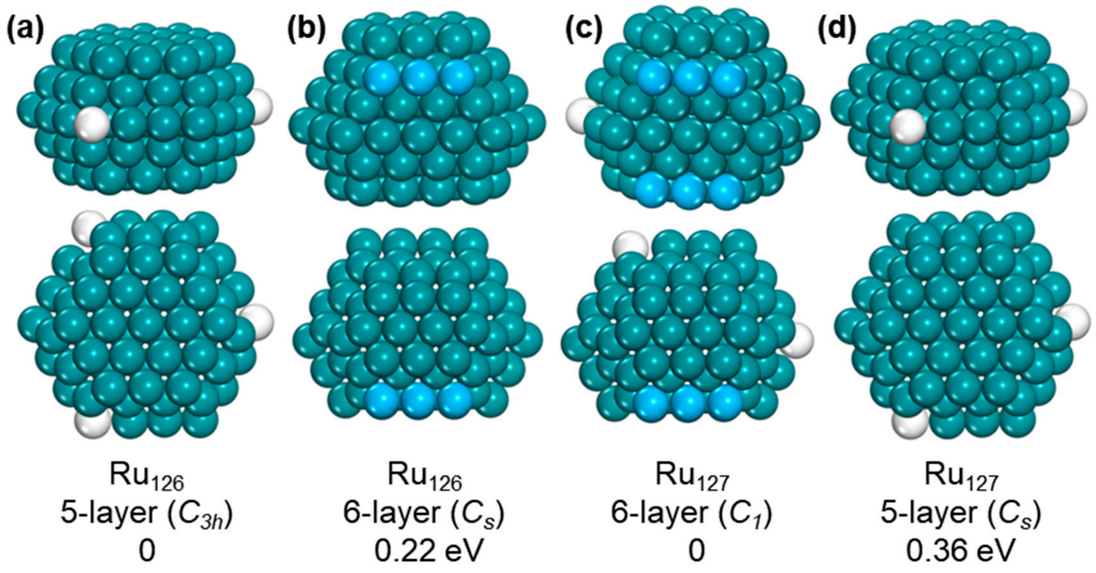

Figure S7. The structure of Ru_{126} and Ru_{127}.

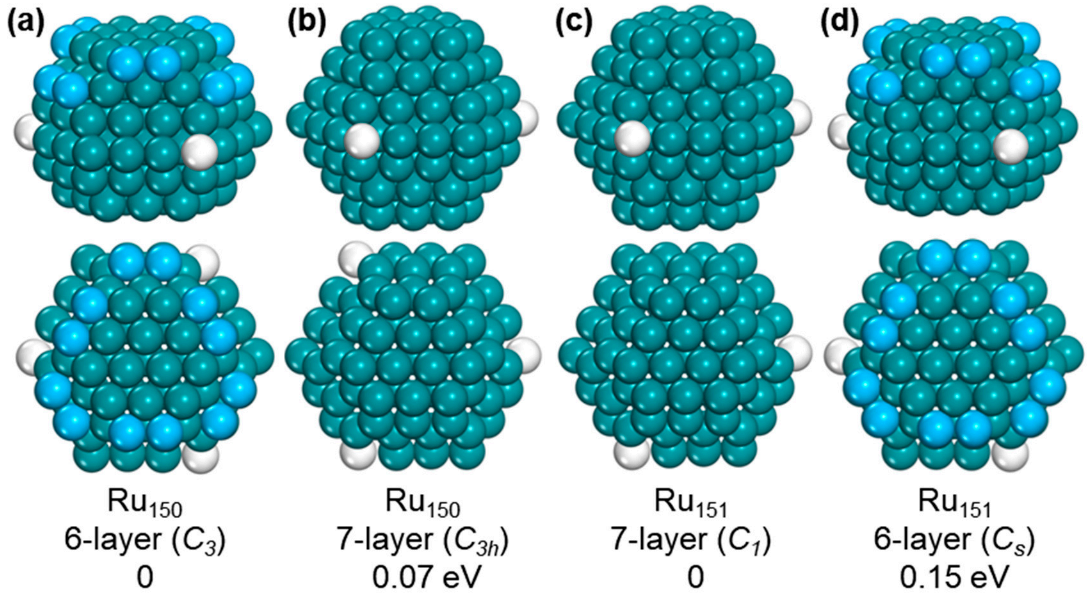

Figure S8. The structure of Ru_{150} and Ru_{151}.

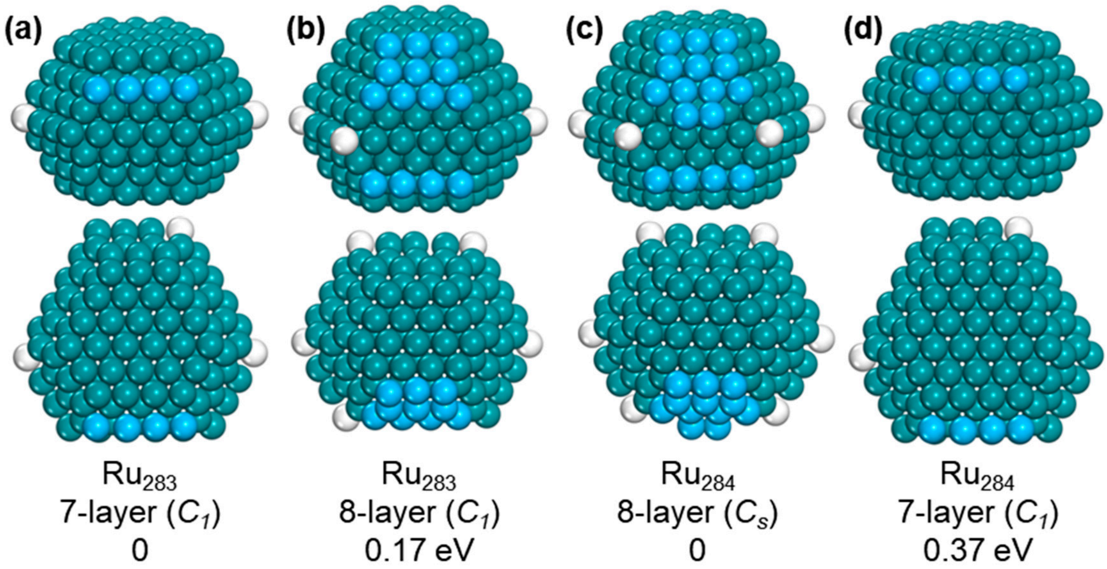

Figure S9. The structure of Ru_{283} and Ru_{284}.

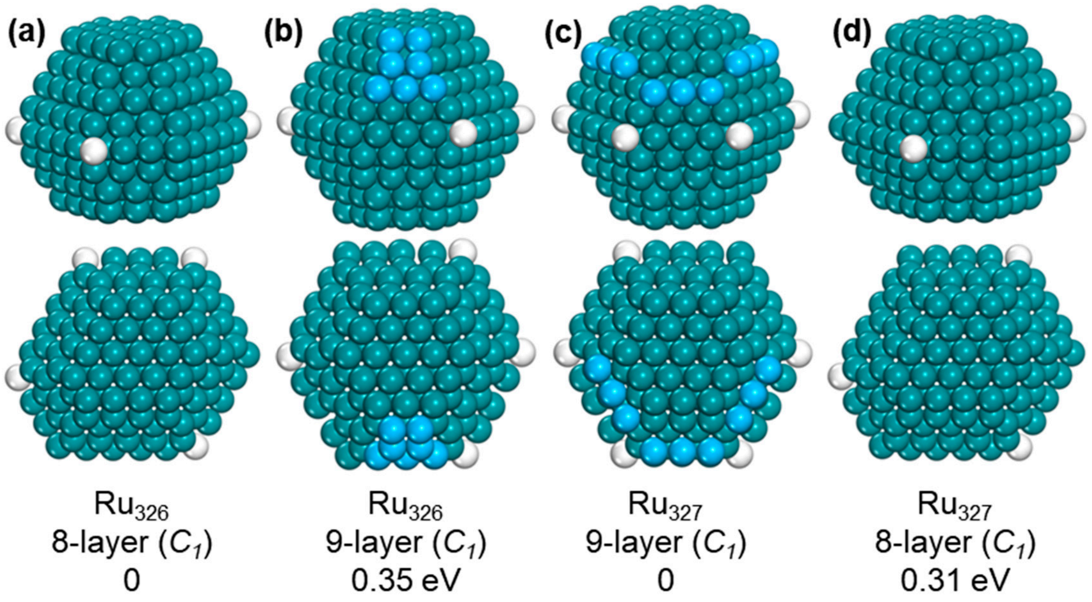

Figure S10. The structure of Ru_{326} and Ru_{327}.

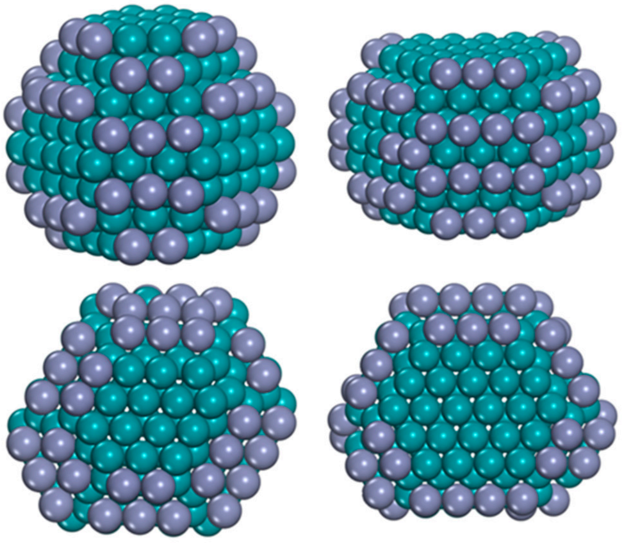

Relative Energy	0	0.19 eV
Average bond length (Ru-Ru and Ru-Zn)	2.650 , 2.617	2.649 , 2.616
Coordination number (Ru-Ru and Ru-Zn)	9.494 , 0.964	9.502 , 0.944

Figure S11. The structure and structural properties of $Ru_{249}Zn_{60}$.

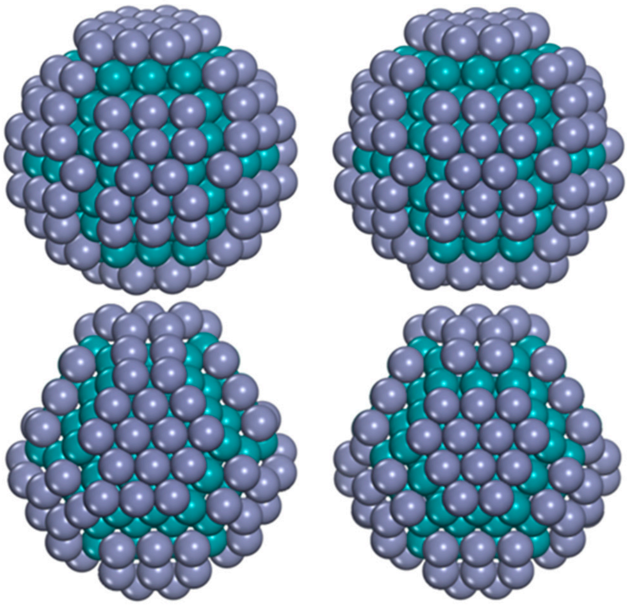

Relative Energy	0	0.25 eV
Average bond length (Ru-Ru and Ru-Zn)	2.657 , 2.643	2.657 , 2.644
Coordination number (Ru-Ru and Ru-Zn)	9.359 , 1.718	9.379 , 1.709

Figure S12. The structure and structural properties of $Ru_{206}Zn_{103}$.

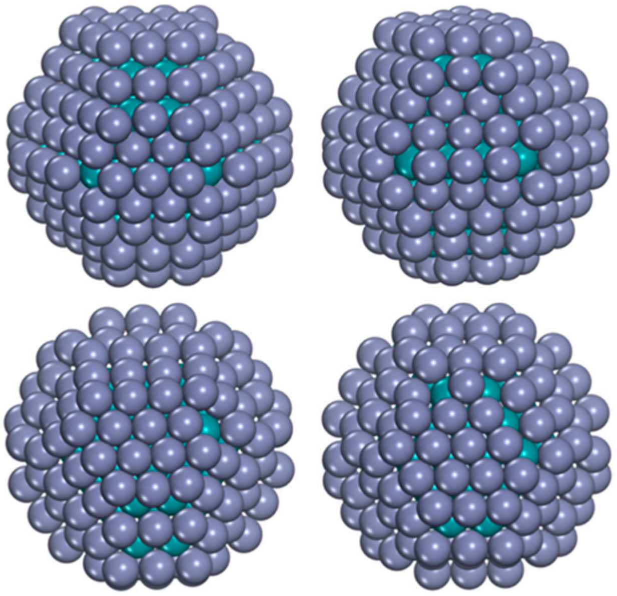

Relative Energy	0	0.34 eV
Average bond length (Ru-Ru and Ru-Zn)	2.669 , 2.660	2.669 , 2.662
Coordination number (Ru-Ru and Ru-Zn)	9.097 , 2.794	9.097 , 2.787

Figure S13. The structure and structural properties of $Ru_{155}Zn_{154}$.

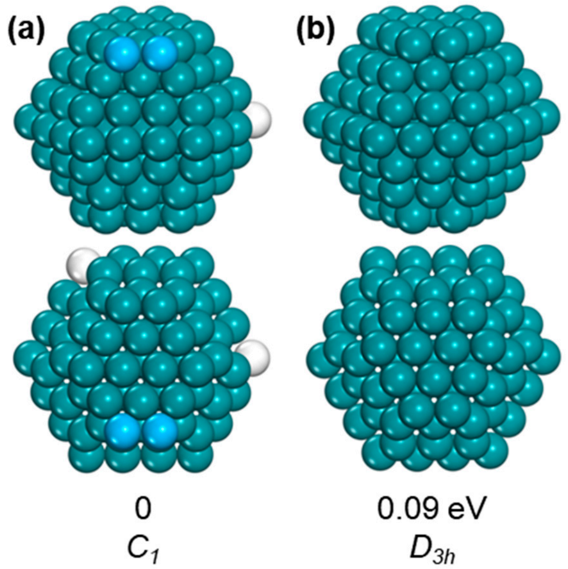

Figure S14. The structure of Ru_{153}.

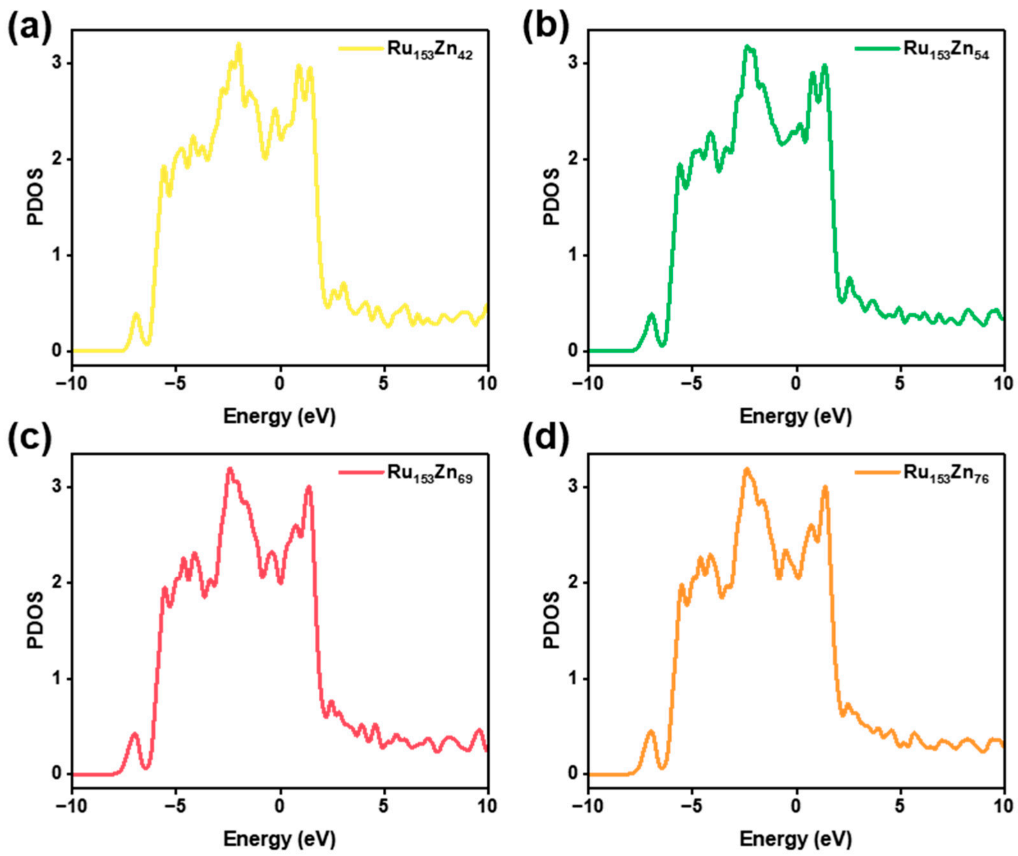

Figure S15. The PDOS of d orbitals for (a) $Ru_{153}Zn_{42}$. (b) $Ru_{153}Zn_{54}$. (c) $Ru_{153}Zn_{69}$ and (d) $Ru_{153}Zn_{76}$.

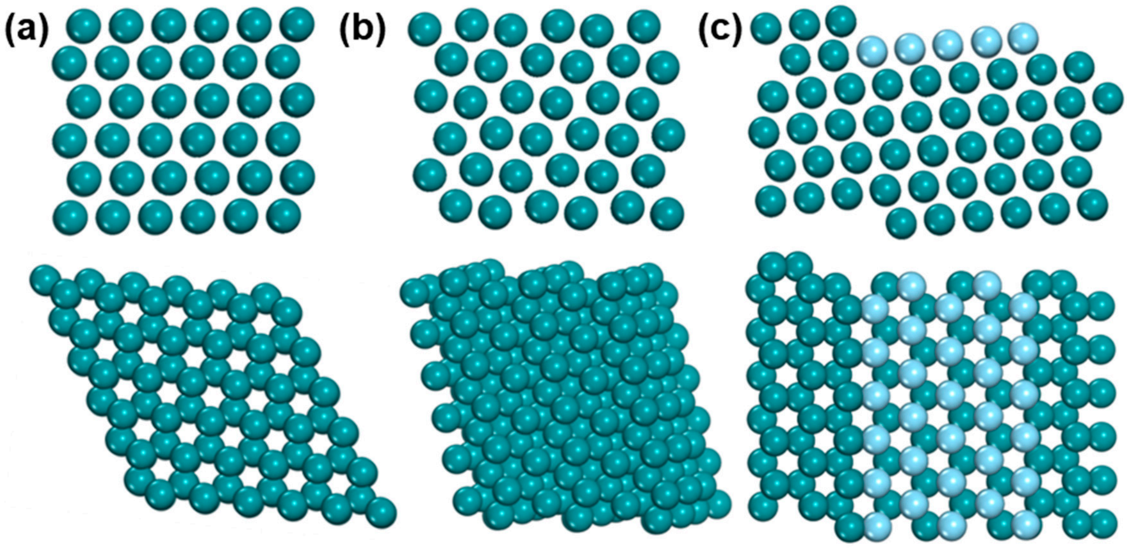

Figure S16. The Ru surface model. The green and blue balls both represent Ru atoms. Side view and top view (a) Ru (0001). (b) Ru $(10\bar{1}1)$. (c) Ru $(10\bar{1}9)$ surface model.

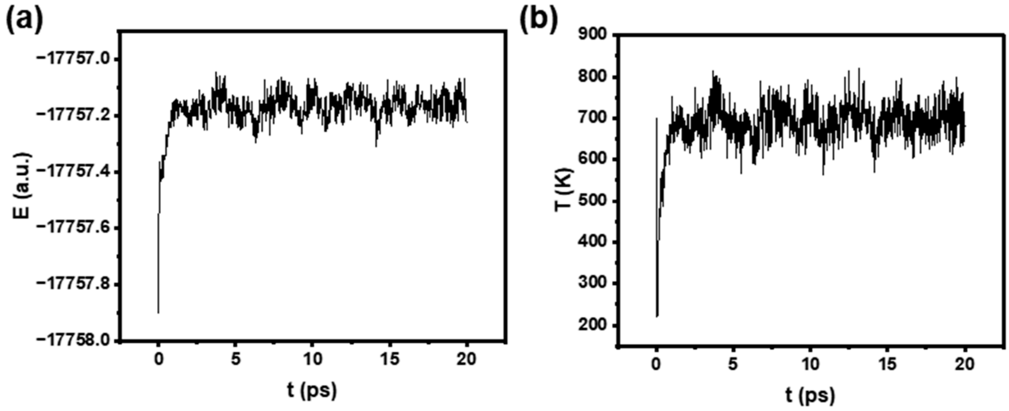

Figure S17. The change of energy and temperature over time in the AIMD simulation, (a) energy and (b) temperature.
